# Relationship between oral health and depression: data from the National Health Survey 2016–2017

**DOI:** 10.1186/s12903-024-03950-2

**Published:** 2024-02-05

**Authors:** Tomás Palomer, Valeria Ramírez, Duniel Ortuño

**Affiliations:** 1 Universidad de los Andes, Santiago, Chile; 2 Pontificia Universidad Católica de Chile, Santiago, Chile

**Keywords:** Oral health, Self-assessment, Mental health, Depression, Health surveys

## Abstract

**Objective:**

To evaluate the relationship between oral health status, self-perception of oral health, and depression.

**Methods:**

This cross-sectional study included 2953 individuals that were ≥ 18 years of age and participated in the Chilean National Health Survey (NHS), 2016–2017. Information on oral, dental, and mental health, and the presence or absence of depressive symptoms was collected. Secondary data analysis was carried out using STATA and included logistic regression models adjusted for sex, age, and educational level. The analyses factored in the expansion weights to estimate representative prevalences of the entire population.

**Results:**

Participants experiencing frequent dental or prosthesis-related discomfort while speaking (OR: 1.57; 95% CI: 1.01–2.43) were related with exhibiting suspected depression. Removable upper denture users were at a higher risk of exhibiting suspected (OR: 2.04; 95% CI: 1.11–3.74) than those not using them. Participants diagnosed with depression in the past 12 months had a similar number of teeth (median = 24) compared to those without depression (median = 25) (OR: 0.99; 95% CI: 0.96–1.02).

**Conclusion:**

Experiencing dental or prosthesis-related difficulties in speaking is related to suspected depression or a diagnosis of depression. These findings highlight the importance of developing comprehensive healthcare approaches that consider mental health in the context of oral health.

**Supplementary Information:**

The online version contains supplementary material available at 10.1186/s12903-024-03950-2.

## Background

In 2019, approximately 970 million people were diagnosed with a mental health disorder. The most common diagnoses were anxiety disorders, affecting 4% of the population, followed by depressive disorders [1]. Neuropsychiatric conditions constitute a significant proportion of the disease burden in Chile, accounting for approximately 23.2% of years of healthy life lost due to disability (YLDs) [2]. Despite rapid progresses in research and access to mental health services, the prevalence of substance abuse and anxiety disorders remain relatively high in the Chilean population [3]. A recent longitudinal study conducted in Chile found that approximately 22.6% and 27.0% of study participants reported moderate to severe anxiety and depressive symptoms in the first and second waves of the COVID-19 pandemic, respectively, suggesting that the levels of mental distress increased between these two time periods [4].

According to the 2019 Global Burden of Diseases report, approximately 3.5 billion people globally live with untreated oral pathologies including dental caries, severe periodontal diseases, tooth loss, and edentulism. Oral pathologies also rank first and third in terms of prevalence and incidence, respectively, and are the tenth most common cause of moderate disability [5]. Similar trends have also been observed in Chile, with the prevalence of oral diseases such as dental caries and periodontal diseases being relatively high in the population [6].

Evidence suggests that individuals diagnosed with mental health disorders are at a higher risk of developing comorbidities due to difficulties associated with seeking and adhering to appropriate treatment plans [7]. Depression is an important risk factor for many systemic conditions including obesity and sleeping disorders. It also plays a significant role in oral health through various biological and behavioral mechanisms, with adoption of risky behaviors such as frequent alcohol consumption, smoking, high fat and sugar intake, and sedentary lifestyles having a negative effect on the patient’s oral health status. Furthermore, the patient’s self-perception of oral health and their frequency of attendance at a dental clinic may also be affected. Previous studies have also reported potential biological mechanisms including an association between depression and reduced salivary flow, xerostomia, and dysregulation of the immune system and salivary immunity. These, in turn, increase the risk of developing oral pathologies such as dental caries and periodontal diseases. As a result, individuals diagnosed with depression typically tend to exhibit a higher prevalence of caries, loss of teeth, and edentulousness [8].

No studies to date have evaluated the relationship between the oral health status, depression, and self-perception of oral health among adults in Chile, and the current study aims to address this gap in knowledge using data from the Chilean National Health Survey (NHS 2016–2017).

## Methods

This cross-sectional study used data from the Chilean NHS 2016–2017; version 3 (Department of Epidemiology, Ministry of Health, Chile); which collected information on the social determinants, related factors, and protective influences of various diseases [6]. The study sample was representative of the Chilean population and included men and women from both rural and urban parts of the country. Pregnant women and individuals who refused to participate in the survey during the home visit were excluded from the study. The survey was carried out using a complex multi-stage clustered, stratified, randomized oversampling technique and had a homeownership rate of 67% and individual participation rate of 90%.

Data collection included home interviews carried out between August 2016 and March 2017 by interviewers and previously calibrated nurses. The survey has 6233 respondents, of which 5520 underwent blood and laboratory testing and oral examination. The first, second, and third visits included interviews; anthropometric measurement and testing (including oral examination) carried out by a nurse; and application of an expanded mental health section to a sub-sample of participants by a trained interviewer, respectively. The oral examination included evaluation of the following items: total number of remaining teeth (both jaws); absence of anterior teeth (yes/no); the total number of teeth with cavitated carious lesions (both jaws); and effective resolution of anterior edentulousness using removable dentures (yes/no; both jaws).

Selected sub-sections (screening, depression, social phobia, agoraphobia, alcohol abuse and dependence, suicidality, mania, psychosis, and use of mental health services) of the Composite International Diagnostic Interview (CIDI), a mental health diagnostic tool developed by the World Health Organization, were applied to a random sub-sample of participants (n = 3403) that were ≥ 18 years of age by a trained interviewer [9]. Older adults who exhibited cognitive impairment during the first visit were excluded. For the extended mental health module application, a random subsample excluded 27 cases that did not meet the inclusion criteria.

The final study sample included 2953(89% of subsample) survey participants that were ≥ 18 years of age. The losses were due to: missing data on the oral health item of interest; failure to undergo oral examination; missing data on the extended mental health section (CIDI); and missing data in the depressive symptoms section.

Depressive symptoms were recorded using an abbreviated version of the CIDI instrument (CIDI Short form; CIDI-SF) containing 30 questions focusing on the presence of dysphoria (sadness symptoms) and anhedonia (lack of interest or ability to enjoy), and a depression risk score was calculated if the patient met at least five out of seven complementary criteria (Diagnostic and Statistical Manual of Mental Disorders or DSM-IV minor criteria for depression).

The participants were diagnosed with depression (as per the CIDI-DSM IV criteria) if they exhibited (1) depressed mood and (2) reduction or loss of interest or pleasure for at least 2 weeks and met ≥ 3 of the following criteria: (1) significant increase or decrease in appetite resulting in substantial weight changes; (2) suicidal ideation; (3) considerable sleep disturbances; (4) psychomotor agitation or motor slow-down; (5) fatigue or loss of energy; (6) feelings of worthlessness or guilt; and (7) decreased concentration.

The last five symptoms must have been experienced all day or almost every day for at least two weeks to be considered in the score. Furthermore, these symptoms must have caused clinically significant discomfort and impairment of social, occupational, and other important aspects of the individual’s life. Therefore, a diagnosis of depression was made if the participant met at least five criteria. Participants with symptoms caused by substance abuse, drugs, medications, and grief or loss of a loved one were excluded.

The variables were defined based on the questions in specific sections of the forms used for corresponding NHS interviews, such as depressive symptoms, oral health, oral examination, and the ‘Depression Section’ (CIDI).

The oral clinical exam, which included third molars, was carried out by trained nurses who participated in a theoretical and practical course with a final test of clinical cases. The interexaminer reliability measured with kappa was 0.85 for tooth loss and cavities presence. Cavities were defined as any surface exhibiting discontinuity, encompassing not only filled teeth but also decayed, temporarily filled, and remaining root structures. Then, the independent variables included the use of dental prosthesis; number of remaining teeth (both jaws); anterior tooth loss; number of decayed teeth; and self-perception of oral health, while the dependent variables were suspected depression and diagnosed with depression in the last 12 months.

The self-perception of oral health was assessed using a five-point ordinal scale. Participants were asked to rate their oral health on a scale ranging from ‘very poor’ to ‘excellent.’ Additionally, specific survey questions focused on oral discomfort and its impact on daily life and social relationships. These questions inquired about discomfort when speaking, pain and suffering, discomfort while eating, interference with daily activities (such as work or study), and interference with social relationships. The responses to these questions provided valuable insights into participants’ overall perception of their oral health and how dental discomfort affected their quality of life.

Descriptive statistics, including percentages for categorical variables and median and dispersion measures for numerical variables, were generated. Logistic regression models were used to estimate OR and 95% CI. Directed acyclic diagrams (not shown) and relationship matrixes (heat plots) were used to examine the association between the variables and outcome measures. The models examining the association between suspected depression and self-perception of oral health were adjusted for sex, level of education, and age, while those exploring the relationship between prosthesis use and the number of remaining teeth were adjusted for the same factors as well as tobacco use. Potential confounding factors considered when examining a diagnosis of depression in the past 12 months as an outcome measure included sex, tobacco, and education, generating open backdoor paths if they do not condition them. The analysis carried out in this study respected the complex sampling and the expansion factors used, which is represented in the results through frequencies and expanded sample sizes. A sensitivity analyses checking the findings robustness using prevalence ratio was performed through generalized linear models with binomial family and log link function. Coefficients from logistic regression model and GLM was compared and tested through adjusted Wald test. All analyses were performed using the statistical software STATA version 16.1 (Windows; STATA Corp. 2019. College Station, TX: StataCorp LLC.).

The NHS 2016–2017 survey was approved by the Scientific Ethics Committee, Faculty of Medicine, Pontificia Universidad Católica de Chile, and informed consent was obtained from all participants. An anonymized version of the database of volunteers has been made available for use for research purposes on the Chilean Ministry of Health website. The current study was approved by the Scientific Ethics Committee of Universidad de los Andes (ID: CEC2021059).

## Results

The study sample included 2953 individuals who participated in the Chilean NHS 2016–2017. Table 1 summarizes patient characteristics by the presence of suspected or diagnosed depression. Approximately 25% of women and 10.53% of men exhibited suspected depression, while 9.84% of women and 2.39% of men had been diagnosed with depression in the past 12 months. Furthermore, the prevalence of a diagnosis of depression in the past 12 months was higher among individuals with higher levels of education (i.e., ≥ 13 years of schooling; 7.26%). Individuals exhibiting suspected depression had a similar median number of teeth (n = 25) while those diagnosed with depression in the past 12 months exhibited a slightly lower median number of teeth (n = 24) compared to those without depression.

**Table 1:** Patient demographics by presence of suspected depression or a diagnosis of depression in the past 12 months (n = 2953)

Variable	Suspected depression			Diagnosis of depression in the past 12 months			
	No	Yes	p-value	No	Yes	p-value	
**Age** (years)	43 (30)	44 (25)	0.1306	43 (28)	48 (26)	0.9284	
**Sex**							
Female	75.00%	25.00%	< 0.0001	90.16%	9.84%	< 0.0001	
Male	89.47%	10.53%		97.61%	2.39%		
**Educational level** (years)							
Less than 8	85.73%	14.27%	0.4954	92.74%	7.26%	0.3337	
8 to 12	81.84%	18.16%		93.29%	6.71%		
13 or more	81.00%	19.00%		95.83%	4.17%		
**Zone**							
Rural	82.52%	17.48%	0.9529	92.84%	7.16%	0.5932	
Urban	82.30%	17.70%		94.07%	5.93%		
**Smoking**							
No	83.80%	16.20%	0.1360	94.64%	5.36%	0.2382	
Yes	79.11%	20.89%		92.39%	7.61%		
**How would you rate your overall oral health?**							
Very good/good	84.06%	15.94%	0.2853	94.47%	5.53%	0.5740	
Regular/Bad/Very bad	80.86%	19.14%		93.47%	6.53%		
**Do your teeth or prostheses cause discomfort when speaking?**							
Never/almost never/sometimes	83.34%	16.66%	0.0398	94.27%	5.73%	0.1671	
Almost always/always	75.30%	24.70%		91.60%	8.40%		
**Do your teeth or prostheses cause pain and suffering?**							
Never/almost never/sometimes	83.27%	16.73%	0.1547	94.69%	5.31%	0.0838	
Almost always/always	78.43%	21.57%		90.8%	9.20%		
**Do your teeth or prostheses cause discomfort when eating?**							
Never/almost never/sometimes	83.59%	16.41%	0.0343	94.62%	5.38%	0.0764	
Almost always/always	76.24%	23.76%		90.64%	9.36%		
**Do your teeth or prostheses interfere with your daily activities (e.g., work, study, housework)?**							
Never/almost never/sometimes	83.00%	17.00%	0.0910	93.98%	6.03%	0.7977	
Almost always/always	74.71%	25.29%		93.46%	6.54%		
**Do your teeth or prostheses interfere with your social relationships?**							
Never/almost never/sometimes	83.15%	16.85%	0.0438	94.15%	5.85%	0.2499	
Almost always/always	73.98%	26.02%		91.72%	8.28%		
**Number of teeth**	25 (9)	25 (9)	0.7776	25 (9)	24 (10)	0.4825	
**Number of teeth with cavitated caries**	1 (2)	1 (2)	0.4336	1 (2)	1 (2)		
**Loss of at least one anterior tooth**							
No	83.25%	16.75%	0.3599	94.68%	5.32%	0.2301	
Yes	80.49%	19.51%		92.43%	7.57%		
**Denture use**							
No	83.34%	16.66%	0.2273	95.18%	4.82%	0.0158	
Upper	74.12%	25.88%	0.0360	87.79%	12.21%	0.0148	
Lower	89.22%	10.78%		92.90%	7.10%		
Upper and lower	86.84%	13.16%		93.75%	6.25%		

Data shown as frequencies expanded (percentages) or medians (75th percentil – 25th percentil diference)

**Fig. 1:**
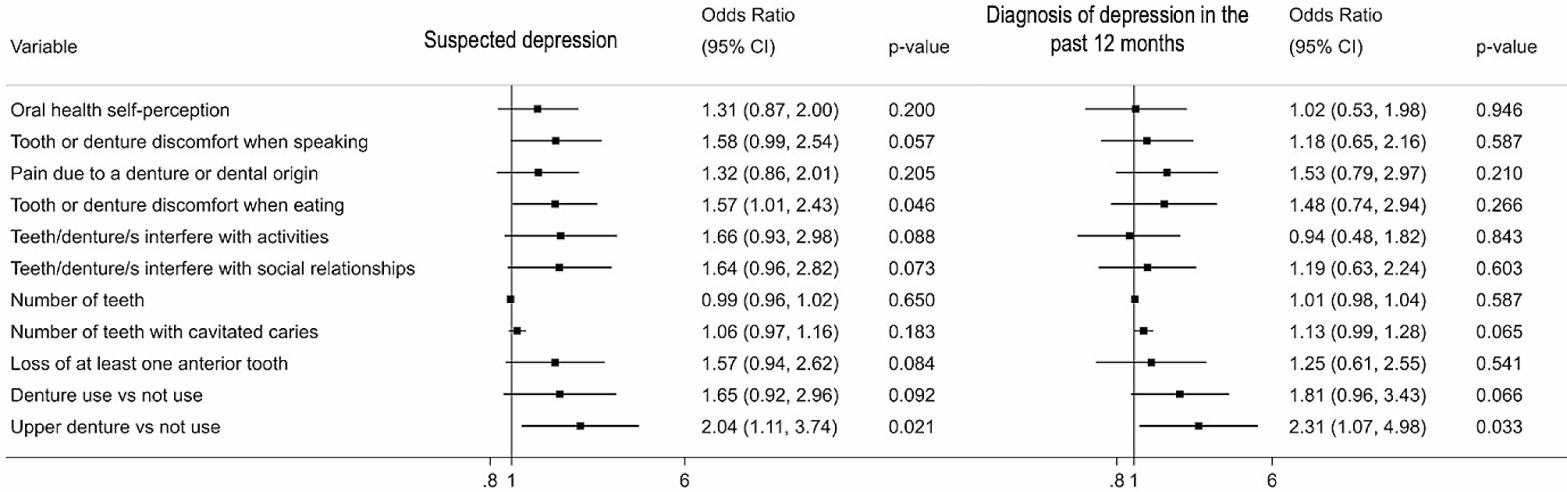
Adjusted logistic regression model showing the relationship between self-perceived oral health, oral health status, suspected depression, and a diagnosis of depression in the past 12 months

**Fig. 2:**
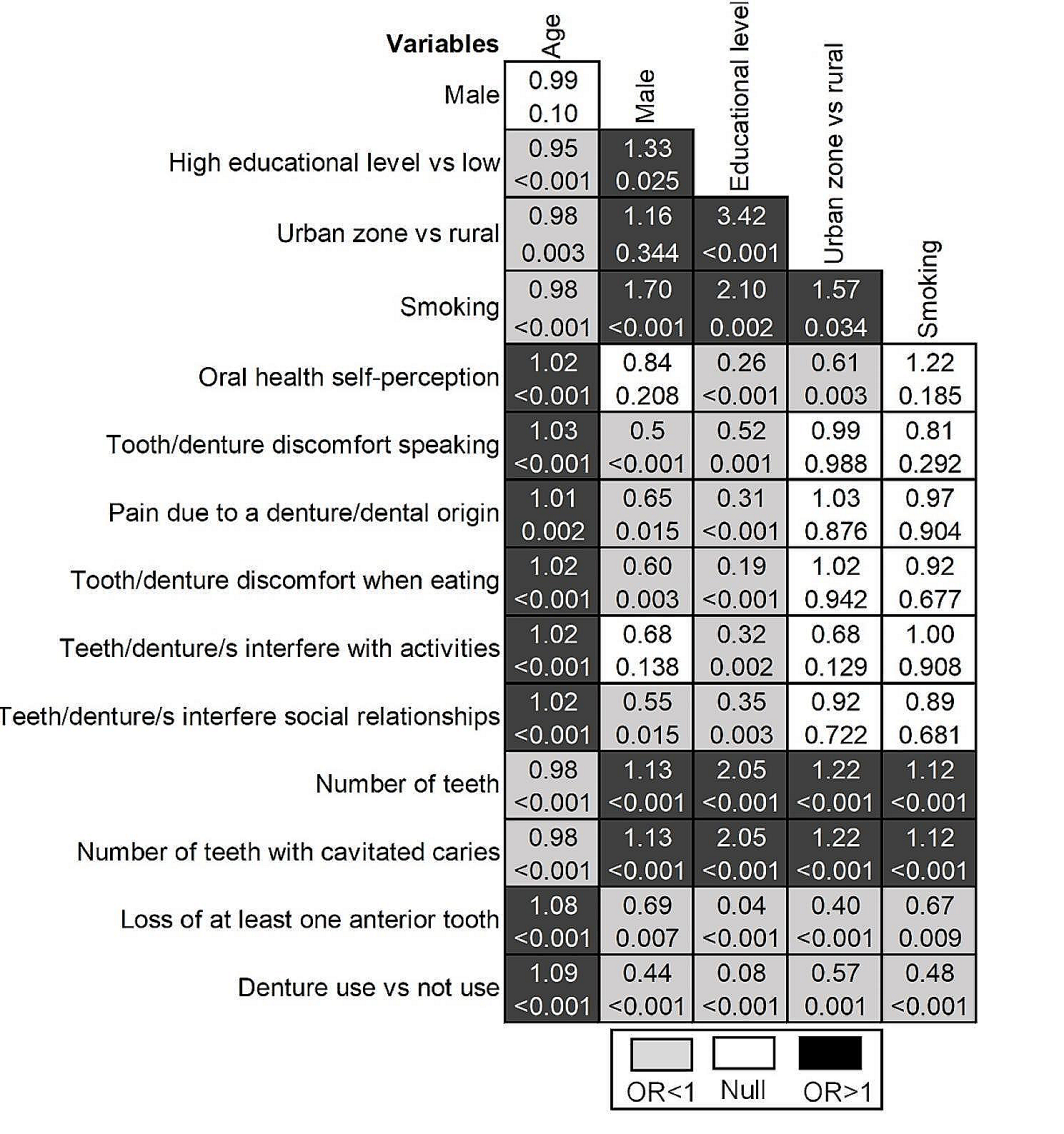
Relationship between variables for identifying factors that contribute to confusion

Figure 1 shows the relationship between oral health, self-perception of oral health, and suspected depression or a diagnosis of depression in the last 12 months. Adjustments were made based on the DAG evaluation, and the relationship matrix has been shown in Fig. 2. The findings showed that patients experiencing difficulties while eating due to dental or prosthesis-related issues were at a higher odds of exhibiting suspected depression (OR: 1.57; 95 CI%: 1.01–2.43) compared to those who did not experience these difficulties. Removable upper denture users were also at a higher odds of exhibiting suspected depression (OR: 2.04; 95% CI: 1.11–3.74) or a diagnosis of depression in the past 12 months when compared to those who did not use prostheses. The results of prevalence ratio as alternative analysis are in Supplementary material, and did not change significatively from the main analysis.

## Discussion

The current study observed a relationship between experiencing dental or prosthesis-related difficulties in speaking, and suspected depression or a diagnosis of depression. Furthermore, participants using removable upper dentures also exhibited higher odds of suspected depression or a diagnosis of depression in the past 12 months.

No significant associations were observed between the number of remaining teeth and depression. This contrasted with several previous cross-sectional or longitudinal studies that reported observing an association between tooth loss and depression, with individuals with fewer remaining teeth being more likely to experience depression. For example, a longitudinal study in the Japanese population found that older adults with fewer teeth were at an increased risk of being diagnosed with depression, potentially due to changes in self-esteem and social support [10]. Another study found that older adults with a higher number of missing teeth were at a greater risk of exhibiting depressive symptoms [11], while Matsuyama et al. [12] showed that losing even one tooth increased the risk of exhibiting depressive symptoms or being diagnosed with major depression. It has been suggested that social factors and oral health mediated this association, with declines in oral function and appearance playing a significant role [13].

The current study observed no significant association between self-perception of oral health and depression or depressive symptoms, and this was in agreement with Kim et al. [14] who concluded that the incidence of depression was higher among individuals who evaluated their oral health using terms such as “poor” or “bad”. Barbosa et al. [15] observed significantly higher (p-value: 0.026) risk of developing depression among individuals with negative self-perceptions of their oral health when compared to those with more positive perceptions (OR: 1.55; 95% CI: 1.05–2.28).

The current study also found that frequent dental or prosthesis-related discomfort while eating was related with a higher frequencie of suspected depression or a diagnosis of depression in the past 12 months. It is important to mention that the presence of oral prosthetics has been related with chewing problems [16] and speaking difficulties and quality of life related with oral health [17]. Park et al. [18] evaluated data from the Korean National Health and Nutrition Examination Survey and found that participants experiencing greater discomfort while eating exhibited a higher risk of depressive symptoms (OR: 1.25; 95% CI: 1.05–1.50) compared to those did not experience such discomfort. Mariño et al. [19] used data from the Melbourne Longitudinal Study on Healthy Aging and found that older Australian adults experiencing oral or dental-related difficulties in eating exhibited significantly higher risk of depressive symptoms (p-value < 0.001) compared to those that did not experience these difficulties, while Kim et al. [14] showed that greater discomfort while chewing or eating was significantly associated with stress, depression, and suicidal ideation. However, discomfort while speaking was only associated with stress but not depression.

Previous studies have also examined the association between denture use and depression, with Seenivasan et al. [20] demonstrating that older adults that used dentures were more likely to experience depression compared to those that did not. Jang [21] compared patients who did and did not use removable dentures and found that the prevalence of depression was 1.07 times higher (p-value < 0.001) in the former group. This could potentially be attributed to emotional and psychological alterations as a consequence of loss of teeth or an inability to adapt to the changes associated with the use of removable prostheses [22]. Tooth loss can trigger depression in vulnerable individuals in particular, and the level of satisfaction with removable prostheses is often determined by certain personality traits [23, 24].

Poor oral health has been shown to be associated with systemic diseases such as depression, with previous studies proposing various underlying biological mechanisms. Oral health problems, particularly those that cause pain, can lead to poor quality of life, stress, anxiety, and depression [25]. Chronic inflammation caused by oral infections, such as periodontitis can also cause alterations in hormonal and neurotransmitter levels in the brain, leading to depression [26]. Finally, poor oral health and tooth loss are often associated with unhealthy dietary habits, reduced nutritional intake, and difficulties while eating, which increases the risk of various mental disorders [27].

This study has several limitations. First, the cross-sectional study design prevented elucidation of causality, with reverse causation remaining a possibility. Second, the study primarily included secondary data analysis which may have affected the results as the data was not collected specifically for this purpose. Third, the oral health examinations were carried out by nurses instead of dentists; however, provision of appropriate training and subsequent calibration ensured high levels of agreement between the examiners, as evidenced in the pilot studies (104). Fourthly, the CIDI-SF instrument does not rule out the possibility of false positives such as chronic diseases, other psychiatric diagnoses (e.g., dysthymia, bipolar disorder, substance abuse), and mourning. Finally, the majority of oral health variables included in this study were self-reported. Future studies may consider examining the relationship between oral health and depression using variables with higher levels of objectivity (e.g., salivary biomarkers).

The key strength of this study was the use of a large study sample that was representative of the Chilean population, ensuring external validity, generalizability, higher statistical power, and reliability of the findings. Finally, the good replicability demonstrated reinforces the robustness of its findings.

## Conclusion

The findings of this study suggest that poor oral health and a negative self-perception of oral health may be related to depression. However, further research is necessary to elucidate the direction of this association, understand the underlying mechanisms involved, and develop effective interventions that adopt a comorbid approach toward improving oral and mental health outcomes.

### Electronic supplementary material

Below is the link to the electronic supplementary material.

Supplementary Material 1. Sensitivity analysis: Odds ratio vs. Prevalence ratio comparison

## Data Availability

This study was nested within the third version of the Chilean National Health Survey (NHS 2016–2017). All NHS 2016–2017 data is freely available through the national repository of population-based surveys carried out by the Ministry of Health (MINSAL, Chile; http://epi.minsal.cl/encuestas-poblacionales/).
