# Correction: Relationship between oral health and depression: data from the National Health Survey 2016–2017

**DOI:** 10.1186/s12903-024-04152-6

**Published:** 2024-03-28

**Authors:** Tomás Palomer, Valeria Ramírez, Duniel Ortuño

**Affiliations:** 1 Universidad de los Andes, Santiago, Chile; 2 Facultad de Odontología, Universidad de los Andes, Santiago, Chile; 3 Pontificia Universidad Católica de Chile, Santiago, Chile

**Correction to: BMC Oral Health (2024) 24:188**

10.1186/s12903-024-03950-2.

In this article [1], the affiliation details for Valeria Ramírez were incorrectly given as “Pontificia Universidad Católica de Chile” but should have been “Facultad de Odontología, Universidad de los Andes, Chile”.

