# Supplementary material for: Relationship between oral health and depression: data from the National Health Survey 2016–2017
Source: BMC Oral Health. 2024 Feb 5;24:188. doi: 10.1186/s12903-024-03950-2 (PMC10845575; doi:10.1186/s12903-024-03950-2)
Supplement: Supplementary file 1 — Supplementary Material 1. Sensitivity analysis: Odds ratio vs. Prevalence ratio comparison [file 12903_2024_3950_MOESM1_ESM.docx]

Supplementary material

| Variable | Outcome | | | | | | | | | |
| --- | --- | --- | --- | --- | --- | --- | --- | --- | --- | --- |
|  | Suspected depression | | | | | Diagnosis of depression in the past 12 months | | | | |
|  | PR | LB | UB | p-value | Comparison p-value | PR | LB | UB | p-value | Comparison p-value |
| Oral health self-perception | 1.25 | 0.9 | 1.73 | 0.189 | 0.2861 | 1.02 | 0.56 | 1.88 | 0.939 | 0.9498 |
| Tooth or denture discomfort when speaking | 1.47 | 1.03 | 2.1 | 0.036 | 0.2282 | 1.16 | 0.68 | 1.97 | 0.596 | 0.5556 |
| Pain due to a denture or dental origin | 1.23 | 0.89 | 1.71 | 0.204 | 0.2473 | 1.46 | 0.81 | 2.63 | 0.205 | 0.2856 |
| Tooth or denture discomfort when eating | 1.43 | 1.03 | 1.99 | 0.034 | 0.1377 | 1.42 | 0.78 | 2.61 | 0.253 | 0.3982 |
| Teeth/denture/s interfere with activities | 1.49 | 0.96 | 2.31 | 0.073 | 0.2156 | 0.94 | 0.52 | 1.72 | 0.850 | 0.8015 |
| Teeth/denture/s interfere with social relationships | 1.48 | 0.98 | 2.23 | 0.061 | 0.1954 | 1.16 | 0.66 | 2.05 | 0.602 | 0.6440 |
| Number of teeth | 0.99 | 0.97 | 1.02 | 0.597 | 0.8597 | 1.01 | 0.98 | 1.03 | 0.547 | 0.8968 |
| Number of teeth with cavitated caries | 1.04 | 0.99 | 1.11 | 0.145 | 0.3120 | 1.10 | 1.01 | 1.2 | 0.030 | 0.2604 |
| Loss of at least one anterior tooth | 1.44 | 0.99 | 2.11 | 0.060 | 0.2672 | 1.22 | 0.64 | 2.32 | 0.546 | 0.5227 |
| Denture use vs not use | 1.55 | 0.97 | 2.47 | 0.065 | 0.3809 | 1.67 | 0.95 | 2.94 | 0.073 | 0.0672 |
| Upper denture vs not use | 1.72 | 1.09 | 2.7 | 0.020 | 0.1920 | 1.99 | 1.05 | 3.78 | 0.035 | 0.0623 |
| PR: Prevalence ratio; LB and UP: Lower and upper bound 95% confidence interval; p-value: GLM model; Comparison p-value: adjusted Wald test | | | | | | | | | | |

Supplementary table. Adjusted generalized linear regression model (GLM) showing the relationship (Prevalence ratio) between self-perceived oral health, oral health status, suspected depression, and a diagnosis of depression in the past 12 months and sensitivity analyses comparing with odds ratio from logistic regression model.
